# Impact of removing cost sharing under the affordable care act (ACA) on mammography and pap test use

**DOI:** 10.1186/s12889-019-6665-9

**Published:** 2019-04-03

**Authors:** Abeer Alharbi, M. Mahmud Khan, Ronnie Horner, Heather Brandt, Cole Chapman

**Affiliations:** 1 Health Services Policy and Management department, Arnold School of Public Health, University of South Carolina, Columbia, SC USA; 2 Health Promotion, Education, and Behavior department, Arnold School of Public Health, University of South Carolina, Columbia, SC USA

**Keywords:** Affordable care act, Cost-sharing, Mammography, Pap tests, Cancer screenings, Preventive care services

## Abstract

**Background:**

The Affordable Care Act (ACA) required private insurers and Medicare to cover recommended preventive services without any cost sharing to improve utilization of these services. This study is an attempt to identify the impact of removing cost sharing on mammography and pap test utilization rates.

**Methods:**

Counterfactual analysis was used to predict what would have been the screening rates in post-ACA if ACA was not there. This was done by estimating a model that examines determinants of dependent variable for the pre-ACA year (pre-ACA year is 2009). The estimated model was then used to predict the dependent variable for the post-ACA year using individual characteristics and other relevant variables unlikely to be affected by ACA (post-ACA year is 2016). Effect of ACA is defined as the difference between the values of dependent variables in post-ACA and the predicted values of dependent variables in the post-ACA year using counterfactual.

**Results:**

The counterfactual analysis show that the utilization of mammogram and pap test did not improve following ACA.

**Conclusion:**

Removal of cost-sharing under the ACA did not improve mammography or pap test rates. Therefore, financial barrier may not be an important factor in affecting utilization of the screening tests and policy makers should focus on other non-financial barriers in order to improve coverage of the tests.

## Background

Cancer is among the leading causes of death in the United States. An estimated 41,400 deaths from invasive breast cancer and 4170 deaths from cervical cancer will occur in 2018 [1]. Mammography and pap test screenings allow early detection of the diseases leading to potentially successful treatment [2–8]. Despite evidence of screening effectiveness in improving health outcomes, rates of mammography and pap test screenings remained suboptimal in the United States [9]. The US Preventive Services Task Force (USPSTF) recommends mammography for women aged 50–74 years every two years, and pap test for women aged 21–65 years every three years [10]. According to the Centers for Disease Control and Prevention (CDC), in 2015, 65.3% of women aged 40 years and older had a mammogram within previous two years, while 69% of women aged 18 years had a pap test within three years [9]. Suboptimal rates of screenings may have resulted from financial barriers women face such as cost-sharing, the amount of money individuals required to pay when seeking medical care. There is evidence that cost-sharing reduces the use of health services, particularly preventive services [11, 12].

The Patient Protection and Affordable Care Act (ACA) required private insurers and Medicare to cover the USPSTF recommended preventive services with a rating of A (strongly recommended) or B (recommended) without any cost sharing. The policy became effective on September 23, 2010 for private health insurers and on January 2011 for Medicare [13]. The goal was to increase the use of preventive services and, in turn, reduce costly events from poorly or unmanaged chronic conditions. Previous research reported mixed results regarding the impact of removing cost sharing on mammography and pap test utilization [14–23]. As the effect of removing cost-sharing on mammography and pap test utilization are still unclear, this study aims to generate evidence on the impact of changes brought about by the ACA on mammography and pap tests rates.

## Methods

### Outcome (dependent variable)

The study outcomes are the self-reported receipt of mammogram and Pap test as measured in the Medical Expenditure Panel Survey (MEPS) dataset. For each preventive service, respondents were asked “About how long has it been since you had this mammogram/Pap test?” with possible responses being “within past year,” “within past 2 years,” etc. In accordance with screening guidelines, a dummy variable was created for mammogram utilization equal to 1 if the test was taken within 1 to 2 years, and a dummy variable for pap test utilization equals to 1 if the test was taken within 1 to 3 years.

### Design

We used a counterfactual analysis to determine the impact of ACA on the preventive screenings rate. Counterfactual analysis helps to understand what would have happened in post-ACA year if ACA was not there. This was done by estimating a model that examines determinants of the dependent variable for pre-ACA (year 2009). Then, the estimated model was used to predict the dependent variable using post-ACA characteristics of individuals (the determinants from the model) (year 2016). The model basically works as pseudo control group allowing estimation of the utilization of screenings if ACA policy changes were absent. Effect of ACA is then estimated as: rate of dependent variable post-ACA minus the predicted rate of dependent variable in the same post-ACA year using counterfactual (that there was no ACA in that year). We chose determinants that we believe may modify the demand for the screening tests and the potential variables incorporated in the model are: age, race, income, education, marital status, region, health insurance type, physical activity, smoking status, comorbidity, routine medical checkup, metropolitan area, out-of-pocket expenses, and the availability of a usual source of care.

It is important to note that there are few determinants of cancer screenings that are likely to change due to the introduction of ACA, implying that incorporating these variables for the post-ACA sample to define the counterfactual will underestimate the effect of ACA. This is because the determinants affected by ACA will pick-up some of the changes happened due to the implementation of ACA. Most important variables likely to change due to the implementation of ACA are coverage rate of health insurance and types of insurance people have. To ensure that the counterfactual estimates are not biased, insurance type and coverage rates were kept the same in the post-ACA year as it was in the pre-ACA year. This was done through adjusting the sampling weights so that pre- and post-ACA insurance coverage and types of insurance coverage are the same. The dollar value of the out-of-pocket variable was adjusted for inflation using MCPI with 2016 as the base year.

### Data

Data for this study was obtained from the Medical Expenditure Panel Survey - Household Component MEPS-HC [24]. The MEPS-HC is a set of large-scale surveys designed and financed by the U.S. federal government to address national questions regarding health care access, utilization, and expenditure in the U.S. It collects data from a sample of families and individuals in selected communities across the United States, drawn from a nationally representative subsample of households that participated in the prior year’s National Health Interview Survey (conducted by the National Center for Health Statistics). During the household interviews, MEPS collects detailed information for each person in the household including health care utilization, health insurance status, coverage source, and out-of-pocket costs. Because the MEPS is the most complete source of data on health care cost, use of health care, and health insurance coverage in the United States, it has been used extensively in scientific publications and published reports, as well as by the Federal and State governments, to examine the delivery and financing of health care in the United States. The combined average response rate for the years 2009 and 2016 was 51.6% [25]. We obtained permission to access the restricted Area Resource File (ARF) that contained a “State” variable from the Agency for Healthcare Research and Quality (AHRQ) which was merged with the publicly available MEPS dataset used in this study. The study was reviewed and approved by the Office of Research Compliance, an administrative office that supports the University of South Carolina Institutional Review Board (USC IRB). All data used was fully anonymized before it was accessed.

### Sample

**Fig. 1:**
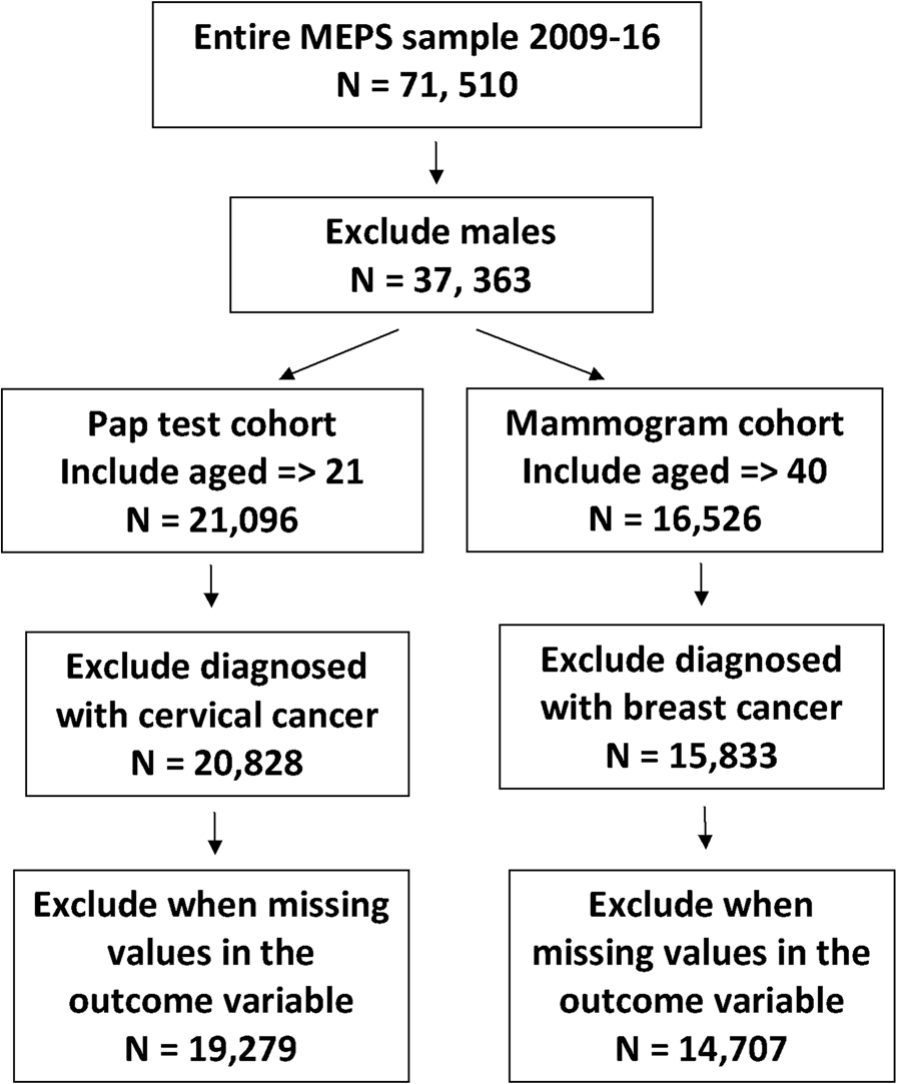
Inclusion and exclusion criteria flow chart

Figure 1 shows the inclusion and exclusion criteria for the study samples. From the MEPS 2009 and 2016 data set, two separate cohorts are prepared to study utilization of mammography and pap test. In accordance with the USPSTF screening guidelines, the mammography cohort includes women aged 40 and older and the pap test cohort includes women aged 21–65. Although the recent USPSTF guidelines regarding breast cancer recommends mammography for women aged 50–74 every 2 years, our mammography cohort included women aged 40 and older because the ACA still utilizes the 2002 guidelines. Women with concurrent or past diagnoses with breast or cervical cancer were excluded from the analysis to focus the analysis on screening for preventive purposes (Fig. 1).

### Statistical analysis

Univariate analyses were done to produce descriptive statistics of women’s characteristics before and after the implementation of ACA in the sample. The main statistical modeling is based on a regression equation explaining the variability of the dependent variables in pre-ACA period using a number of determinants or explanatory variables. The equations estimated can be written as:

*Y*_*i*0_ = *β*_0_ +  ∑ *β*_*j*_*X*_*ij*0_, where Y_io_ is the value of dependent variable for individual i for the pre-ACA year 0 and [Xj] is a set of potential determinants of Y. This estimated model was then used to predict the dependent variable for the post-ACA years using the values of determinants in the post-ACA data set. In other words, we have predicted the values of Y for the post-ACA year t using the estimates of β from the pre-ACA year.

$$ {Y}_{it}=\widehat{\beta_0}+\sum \widehat{\beta_j}{X}_{ijt} $$

Another regression model was estimated to predict the values of Y in post-ACA year using post-ACA data. The effect of ACA would be the value of dependent variable in the post-ACA year minus the predicted value of the dependent variable in the post-ACA year using the regression model obtained using pre-ACA year (the counterfactual). A positive difference means that women in post-ACA used more mammography and pap tests compared to pre-ACA year given various determinants of the dependent variable. Since a number of potential determinants of dependent variable may be affected by the ACA-triggered changes, these variables should be kept constant at the pre-ACA level to obtain the true effect of ACA. These variables are related with insurance coverage and types of insurance under which the individuals are covered. We have kept the values of these determinants constant in relative terms at the pre-ACA level by changing the sampling weights proportionately. To assess the diagnostic/predictive accuracy of our logistic model, we used the area under the ROC curve measure.

To better understand the estimated effects of ACA, we regressed the difference of the dependent variables on some population characteristics to examine how different individual characteristics affect the outcomes. Differences were considered statistically significant if *P*-value of the t-statistics < 0.05. All statistical analyses were carried out using STATA software version 14 (2015; Stata 14.0 Statistical Software, College Station, TX, USA The analyses accounted for probability weighting in the MEPS [26, 27].

## Results

**Fig. 2:**
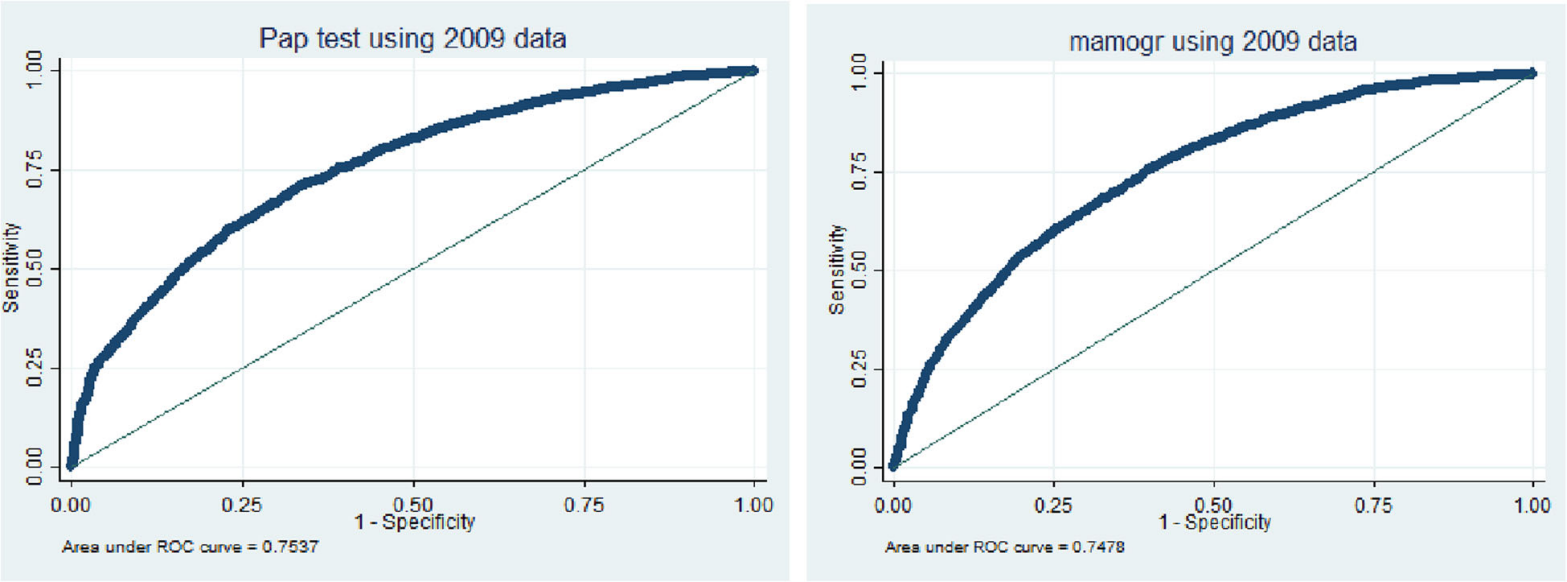
Area under the ROC curve for the estimating models

Table 1 shows descriptive statistics of the women’s characteristics in pre and post ACA. Women in pre-ACA and post-ACA seem to have similar distribution demographic characteristics including age, income, education, race, and insurance status, and the availability of usual source of care (Table 1). Figure 2 shows the results from the area under the ROC curve measure for our estimation models which indicate that both estimating models had an area under the curve was above 74%. Tables 2 shows the counterfactual analysis results which found that the utilization of mammogram and pap test did not increase following the ACA. Women in post-ACA used less mammography and pap tests than the same period post-ACA counterfactual (Table 2). The difference for mammography is − 2.31 and for pap test is − 5.65 (Table 2). Tables 3 and 4 breaks down those differences by age, race, income, education, and health insurance type. A positive difference indicates that this particular group decreases the difference while a negative difference indicate that this particular group increases the difference. For most categories, the difference was negative except for women aged 50–64 and women with public insurance implying that women aged 50–64 or with public insurance used higher level of mammogram and pap tests in post-ACA compared to the pre-ACA levels (Table 3). Similarly, women used less pap tests in all sub-groups in post-ACA year compared to the level in counterfactual estimates (Table 4). Table 5 shows that difference in probabilities are statistically significant.

**Table 1:** Descriptive statistics of the characteristics of women aged > = 21 in pre-ACA (2009) and post-ACA (2016), MEPS database (Results)

Characteristics		Pre-ACA *n* = 13,146	Post-ACA *n* = 12,786
		%	%
Age	Mean	47	48
Race	White	68	68
	Black	21	20
	Other	9	11
Education	Some school	21	19
	High School	31	29
	College	47	50
Income	Low	42	42
	Middle	29	27
	High	27	29
Insurance	Private	57	57
	Public	24	30
	Uninsured	18	11
Usual source of care	Available	77	77
	Not available	22	22

**Table 2:** The difference in mammography and Pap tests use between post-ACA and post-ACA counterfactual (Results)

	Post-ACA counterfactual Predicting probabilities using 2009 model	Post-ACA Predicting probabilities using 2016 model	Difference
Mammogram	71.67%	69.36%	−2.31
Pap test	79.42%	73.77%	−5.65

**Table 3:** The difference in mammography use between post-ACA and post-ACA counterfactual, by different population groups (Results)

Category	Post-ACA counterfactual Predicting probabilities using 2009 model	Post-ACA Predicting probabilities using 2016 model	Difference
Low income	62.29%	60.62%	−1.67
Middle income	71.15%	69.82%	−1.33
High income	82.40%	78.56%	−3.84
Some school	60.61%	57.23%	−3.38
High school	68.31%	68.08%	−0.23
College	78.33%	75.21%	−3.12
White	70.56%	68.46%	−2.1
Black	76.24%	74.73%	−1.51
Other	70.73%	65.57%	−5.16
40–49	65.50%	60.96%	−4.54
50–64	74.98%	75.52%	+ 0.54
> = 65	72.49%	69.38%	−3.11
Private insurance	77.70%	74.92%	−2.78
Public insurance	66.64%	66.70%	+ 0.06
Uninsured	42.75%	39.50%	−3.25
Usual Source of Care available	76.03%	72.82%	−3.21
Usual Source of Care not available	45.33%	49.96%	−4.63

**Table 4:** The difference in Pap tests use between post-ACA and post-ACA counterfactual, by different population groups (Results)

Category	Post-ACA counterfactual Predicting probabilities using 2009 model	Post-ACA Predicting probabilities using 2016 model	Difference
Low income	76.24%	70.22%	−6.05
Middle income	78.52%	72.68%	−5.84
High income	84.85%	79.92%	−4.93
Some school	70.46%	66.56%	−3.9
High school	75.76%	69.22%	−6.54
College	84.94%	79.14%	−5.8
White	78.16%	71.62%	−6.54
Black	85.32%	81.48%	−3.84
Other	76.71%	73.46%	−3.25
21–39	89.26%	86.17%	−3.09
40–49	86.04%	84.51%	−1.53
50–64	78.29%	73.74%	−4.55
Private insurance	83.52%	79.09%	−4.43
Public insurance	73.89%	65.44%	−8.45
Uninsured	73.13%	68.96%	−4.17
Usual Source of Care available	86.84%	83.77%	−3.07
Usual Source of Care not available	79.18%	75.47%	−3.71

**Table 5:** Likelihood of the difference between post-ACA and post-ACA counterfactual to increase/decrease explained by different determinants (Results)

Category	Difference in mammogram	Difference in Pap test
Low income	0 (.)	0 (.)
Middle income	0.0256*** (37.05)	0.000804 (0.95)
High income	−0.00406*** (−5.58)	0.0345*** (34.70)
Some school	0 (.)	0 (.)
High school	0.0298*** (37.44)	− 0.0204*** (−19.26)
College	0.0120*** (15.32)	−0.0280*** (−28.37)
White	0 (.)	0 (.)
Black	−0.0196*** (−24.37)	0.0249*** (33.66)
Other	−0.0386*** (−38.73)	0.0155*** (17.02)
21–39		0 (.)
40–49	0 (.)	0.00758*** (9.81)
50–64	0.0554*** (67.09)	−0.0303*** (−35.82)
> = 65	0.0284*** (32.22)	
Private insurance	0 (.)	0 (.)
Public insurance	0.0285*** (42.36)	−0.0187*** (−20.24)
Uninsured	−0.0279*** (−19.43)	− 0.0191*** (−13.70)
Cons_	− 0.0651*** (−36.92)	−0.0149*** (−8.80)
N	6364	8924

t statistics in parentheses * *p* < 0.05, ** *p* < 0.01, *** *p* < 0.001

## Discussion

Because cost-sharing was found to reduce the use of medical care services, particularly preventive care services, the ACA required private health insurers and Medicaid to cover preventive screenings recommended by the USPSTF without cost-sharing. This study used a counterfactual analysis to try to understand if the ACA’s cost-sharing provisions impacted mammograms and Pap tests rates.

In the years preceding the ACA, both mammogram and Pap test coverage rates showed declining trends among women in the U.S. [9]. Our results show that the introduction of free preventive services did not affect the overall declines in mammograms and Pap tests. These results are consistent with previous studies that found little impact of cost-sharing provision on mammography and pap tests rates. For example, Hong et al. [23] found decreased rates of being up-to-date on mammograms and Pap tests among those with private insurance after the ACA. Several other studies examined the initial impact of removing cost-sharing on privately insured women and found no change in mammography and pap test [17–19]. Our results show no impact on mammography use among women with Medicare (Table 3). Previous studies that looked at Medicare population obtained mixed results regarding mammography utilization after the ACA. For example, Jensen et al. [21] found minimal change in mammography use among older women, while other studies found statistically significant increase in mammography uptake after the ACA [14, 15]. Our analysis shows positive results in mammogram rates for women aged 50–64 (Table 3). This is consistent with a previous study that assessed the policy from a health system level and found that ACA provisions were associated with increased screening volumes among 50–74 year old women [22]. There is evidence that mammogram decrease breast cancer mortality among women aged 50–69 but the benefit of mammography for women aged 40–49 years is uncertain [28, 29]. Our results show that younger women used more Pap tests than older women (Table 4). This is consistent with the guidelines and the evidence suggesting benefits from Pap tests are more evident for younger women [30].

Our results show that black women had the lowest decline in mammography and women from other minorities had the lowest decline in Pap test use compared to white women (Table 3 and Table 4). A recent study found the ACA provision to be associated with improved mammography and pap tests among Hispanics and African Americans [31]. Looking at overall utilization, black women had the highest rate of mammogram and pap tests use (mammogram: 81.48%, pap tests: 74.73%) (Table 3 and Table 4). Evidences show that black women in the U.S. are more likely to be diagnosed and die from breast and cervical cancer than the white women, which may explain the increased use of screenings among this group [32, 33].

There are some possible explanations for the little impact of the ACA cost-sharing provision on slowing or reversing the declining rates of mammography and pap tests. First, it is important to recognize that ACA’s provision of no cost-sharing for preventive services was found to be effective in improving coverage of a number of preventive services such as blood pressure check, cholesterol check, and flu vaccination, but it had no effect on cancer screenings. This is probably because the guidelines for mammograms and Pap tests were updated around the time of the ACA’s provisions. The guidelines for mammograms were updated in 2009 to recommend mammograms for women aged 50–75 every 2 years updating the previous guidelines that recommended screening every 1–2 years for women aged 40 and older. The guidelines for cervical cancer screenings was updated in 2012 to recommend the Pap test for women aged 21–65 every three years updating the previous guideline of screening annually for women who are sexually active. These guideline updates, which recommended reductions in the frequency of the tests, may have led to overall declines in these cancer screenings. In addition to the changing guidelines, some women find the tests toxic or painful which may discourage utilization [34–37]. Sometimes mammograms yield false positive cases implying that some perfectly healthy women will go through the pain, anxiety, and other side effects because of the treatment. Similar to mammograms, Pap tests may come with harm when abnormal results lead to vaginal bleeding, pain, infection, and anxiety. Physicians make trade-offs between benefits and risks when making recommendations about who should be screened.

The second reason for little or no impact of ACA provision could be that mammography and Pap tests were covered with no or minimum cost-sharing by some private insurance plans under Medicare Advantage even before the introduction of ACA. The amount of cost-sharing under Medicare was relatively small anyway prior to ACA (20% cost sharing) and the new ACA provision may not have affected out-of-pocket expenses significantly. Another possibility is that some women obtained screenings through national programs like CDC’s National Breast and Cervical Cancer Early Detection Program (BCCEDP) in both pre- and post-ACA years. The program was established in 1990 to provide free and/or reduced cost mammograms and pap test to women with limited incomes and those who lacked health insurance. The program continued to provide large number of screenings in the years following the ACA [38]. Any change in the coverage of the program between pre- and post-ACA years may show up as lack of effect of ACA unless screenings done through these programs are controlled for. Unfortunately, due to lack of data, it was not possible to exclude women who obtained the cancer screenings through the BCCEDP.

In the years before the ACA, low-income women were less likely to receive lifesaving recommended cancer screenings [39]. A new study found that the ACA was associated with improvements in health care-related financial strain [40]. However, socioeconomic disparities remained in term of mammograms and Pap tests utilization. In our cohort, the majority of screenings occurred among the high-income high-educated women (Table 3 and Table 4). This is probably due to better health literacy, awareness, and availability of time and transportation. Our analysis show that the proportion of women who reported having a usual source of care used more mammogram and Pap tests than those with no usual source of care (Table 3 and Table 4). Therefore, factors related to provider availability and access may be more effective in improving utilization rates.

Finally, it is important to recognize that, although screening numbers did not improve following the ACA, disease burden from breast and cervical cancer has been declining over the years. Breast cancer rates have been declining steadily in the past decade. Cervical cancer cases have declined rapidly as well in past 40 years due to wide use of Pap tests. Declines in cervical cancer cases, however, have slowed down in recent years.

## Conclusion

The rates of mammograms and Pap tests were already in a declining trend in the years before the ACA and the introduction of ACA provision of no cost-sharing did not help change the declining trend. The rationale behind the ACA was built on the notion that cost-sharing hinders the use of preventive care services. Several post-ACA studies found positive impact of the ACA on improving certain kinds of preventive care services but not cancer screenings. Absence of positive effects of ACA on cancer screenings is possibly due to the changes in guidelines for mammograms and Pap tests and/or due to the fear of side effects from these screenings. Although the overall screenings declined following the ACA, declines were the lowest among women from minority groups for Pap tests and the lowest among black women for mammograms which indicate reducing screening disparities by race. In addition, mammograms rates improved among women aged 50–64 – the age group most likely to benefit from the screening. Finally, the results indicate – consistent with previous studies - that the higher level of utilization rates are observed among the wealthiest and most educated women even after the services became free after the policy change. It appears that financial barrier was not the most important factor in affecting utilization of these screening tests. Therefore, policy makers should focus efforts on facilitating access, health promotion, and awareness which may help improve screening rates. Future research is recommended to look at possible factors affecting access to care, provider availability and characteristics, physician compliance to guidelines to better understand the reasons for lack of effects of cost reductions on utilization of cancer screenings.

We acknowledge several limitations of this study. First, information about outcomes relied on self-reported survey responses which might be subject to recall error. However, the MEPS follow up with health providers to reduce measurement errors but some errors may remain, especially for procedures and tests requiring longer recall. Second, the data used in the analysis are cross-sectional and comparison of cross-sectional data of different years is not same as observing changes in the outcomes of the same set of individuals over the years with the implementation of the ACA. The study design made an attempt to tease-out the effect of policy changes. Third, a longer time frame may be needed to be able to see the effects of the policy changes on these outcomes. Fourth, for the privately insured population, there is no information on the coverage of screenings as health plans that were grandfathered were not subject to the ACA provision. Because the elimination of cost-sharing is not universal for the privately insured, full effect of removal of cost sharing will not be observable for this population group.

Another important factor for the lack of positive effect of cost-sharing removal may be due to changes in the USPSTF guidelines for breast cancer and cervical screening that occurred around the same time as the introduction of ACA provisions. The change in the guidelines may have led to overall declines in cancer screening. In 2009 the guidelines for breast cancer screenings was updated to recommend biennial screening instead of every 1–2 years. Also, in 2012 the USPSTF guidelines for cervical cancer was updated to recommend the test every 3 years instead of every year. If the physicians start using the new guidelines, mammogram and Pap test screenings may appear lower if prior guideline based utilization rates are calculated and compared. Counterfactual analysis will not be able to correct for the changes in screening guidelines unless a control group can be identified for comparative purposes.

In any case, the analysis raises the concern that implementation of ACA’s cost share removal has not been effective in improving cancer screening rates and longitudinal survey data would be needed to understand why the removal of out of pocket costs failed to show the intended effects. Unfortunately, the data we have are repeated cross-sectional. Longitudinal data covering a period of four to five years are not available to conduct an analysis to find out how the utilization of cancer screening tests changed for the same individual over the years.

## Abbreviations

ACA: Affordable Care Act
CDC: Center for Disease and Control
MEPS-HC: Medical Expenditure and Patient Services – Household Component
POST-ACA: After the Affordable Care Act
PRE-ACA: Before the Affordable Care Act
USPSTF: U.S. Preventive Services Task Force
